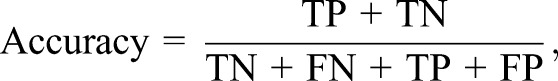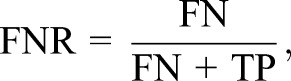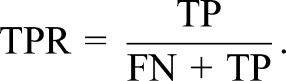# Changes in cell shape are correlated with metastatic potential in murine and human osteosarcomas

**DOI:** 10.1242/bio.013409

**Published:** 2016-02-12

**Authors:** Samanthe M. Lyons, Elaheh Alizadeh, Joshua Mannheimer, Katherine Schuamberg, Jordan Castle, Bryce Schroder, Philip Turk, Douglas Thamm, Ashok Prasad

**Affiliations:** 1 School of Biomedical Engineering, Colorado State University, Fort Collins, CO 80523, USA; 2 College of Veterinary Medicine and Biomedical Sciences, Colorado State University, Fort Collins, CO 80523, USA; 3 Department of Chemical and Biological Engineering, Colorado State University, Fort Collins, CO 80523, USA; 4 Department of Biology, Colorado State University, Fort Collins, CO 80523, USA; 5 Department of Statistics, Colorado State University, Fort Collins, CO 80523, USA

**Keywords:** Cell shape, Cytoskeleton, Invasiveness, Machine learning, Metastasis, Morphometrics

## Abstract

Metastatic cancer cells for many cancers are known to have altered cytoskeletal properties, in particular to be more deformable and contractile. Consequently, shape characteristics of more metastatic cancer cells may be expected to have diverged from those of their parental cells. To examine this hypothesis we study shape characteristics of paired osteosarcoma cell lines, each consisting of a less metastatic parental line and a more metastatic line, derived from the former by *in vivo* selection. Two-dimensional images of four pairs of lines were processed. Statistical analysis of morphometric characteristics shows that shape characteristics of the metastatic cell line are partly overlapping and partly diverged from the parental line. Significantly, the shape changes fall into two categories, with three paired cell lines displaying a more mesenchymal-like morphology, while the fourth displaying a change towards a more rounded morphology. A neural network algorithm could distinguish between samples of the less metastatic cells from the more metastatic cells with near perfect accuracy. Thus, subtle changes in shape carry information about the genetic changes that lead to invasiveness and metastasis of osteosarcoma cancer cells.

## Abstract (precis)

**Summary:** Human and murine invasive osteosarcoma cancer cell lines, developed by selection *in vivo* from a less invasive parental line, show distinguishable differences in shape from the parental line that fall into two categories: more mesenchymal or more amoeboid.

## INTRODUCTION

Despite significant advances in treatment of cancer, it remains the leading cause of death in both men and women under 80 years of age in the US (Siegel et al., 2014), with metastasis as the cause of 90% of human deaths from cancer (Gupta and Massague, 2006; Siegel et al., 2014). Understanding and prevention of cancer invasion and metastasis is key in reducing cancer mortality (Gupta and Massague, 2006). Multiple studies have pointed out that the acquisition of invasiveness appears to require changes in mechanical properties of cancer cells, which may be linked to the functional properties that are necessary for metastasis (Makale, 2007; Suresh, 2007). To form successful metastases, tumor cells must navigate a complex, multi-stage process including: detachment from primary tumor, migration to vascular supply, intravasation, survival and transit in blood or lymphatic vessels, extravasation, and successful growth and adhesion in a new site (Chambers et al., 2002). Metastatic cells have been found to be softer or more deformable than non-metastatic cells in analysis with atomic force microscopy (Cross et al., 2007, 2008; Li et al., 2008; Xu et al., 2012) and optical lasers (Guck et al., 2001, 2005; Runge et al., 2014). In addition to cell deformability, multiple studies have shown that molecules responsible for cell-extracellular matrix and cell-cell adhesion interactions, including cadherins and integrins, are down-regulated or altered in cancer cells (Berx and van Roy, 2009; Cavallaro and Christofori, 2004; Chen et al., 2013; Hanahan and Weinberg, 2011; Rathinam and Alahari, 2010). Cancer cell deformability is linked with invasiveness and can be an indicator of metastatic potential (Kenny et al., 2007; Paszek et al., 2005; Suresh, 2007; Tullberg and Burger, 1985). However softness is just one aspect of cellular biomechanics. Cells are active objects and can exert contractile forces on the extracellular matrix; there are some reports that more invasive cells are more contractile (McGrail et al., 2015). Understanding and identifying altered biomechanical properties of aggressive cancer cells can provide crucial information for assessing the invasiveness of cancer cells.

Direct assays of mechanical changes require fairly complex and expensive instrumentation. However, one can hypothesize that changes in biomechanical properties, including changes in cytoskeletal properties and expression of adhesion proteins, would translate into changes in cell shape. It has been shown previously that changes in gene expression of genes with cytoskeletal function leads to shape changes that can be detected using morphometric characteristics (Bakal et al., 2007). Cytoskeletal gene expression changes that are signatures of metastatic capacity therefore may be detectable by morphometric analysis. Ability to detect such changes would be of great use in assessing cancer clinically.

One of the gold standards of predicting clinical outcome of cancer is tumor grading which includes assessment of cellular morphology from tumor tissue samples. Tumor grading schemes focus on overt changes in cellular morphology such as mitotic index, degree of nuclear pleomorphism and degree of tumor necrosis (Kirpensteijn et al., 2002; Straw et al., 1996). What is not known is whether morphometric parameters of the two-dimensional shape of the cell are sensitive to the changes in cellular properties that accompany the acquisition of invasiveness.

Our paper is based on the hypothesis that subtle changes in cellular properties should manifest themselves in small but detectable morphological changes because of the importance of cytoskeletal changes for acquisition of invasive capacity. The biomechanical changes that accompany the emergence of aggressive tumor cells should be detectable by assaying the changes in shape of these tumor cells. Moreover, the observation of specific changes in shape may be linked with specific genetic changes in cancers.

Anecdotal evidence for the change in cell shape has been well documented. For example, the epithelial to mesenchymal transition (EMT), associated with development of the invasive phenotype in carcinomas (Kalluri and Weinberg, 2009), is often accompanied by acquisition of a mesenchymal-like elongated spindle morphology (Cowden Dahl et al., 2009; Odenwald et al., 2013). Studies have found that tumors which have formed metastases at the time of diagnosis have significantly higher grades, and thus grossly altered morphology, than non-metastatic osteosarcomas (Loukopoulos and Robinson, 2007). An understanding of the relationship between cell shape and the invasiveness of the cancer would lead to a deeper understanding of the relationship between carcinogenic transformation and shape regulation and may allow for a more accurate assessment of cancer outcome from cancer biopsies. Assays that can reliably estimate the percentage of potentially invasive cells in a heterogeneous sample of primary tumor cells may be of great value for guiding therapeutics.

We utilized osteosarcoma cell lines due to the high metastatic rate of the cancer. Osteosarcoma (OSA) is the most common primary bone tumor of dogs (Morello et al., 2011) and humans (Ottaviani and Jaffe, 2009). OSA has a high rate of metastasis and routinely forms metastases to the lung, often before the primary tumor is diagnosed and more than 80% of human OSA patients have metastases at the time of diagnosis (Kaste et al., 1999; Link et al., 1986; Ward et al., 1994; Yonemoto et al., 1998), most with pulmonary metastases (Jaffe et al., 2003; Kager et al., 2003).

Comparing the morphology of cells that were closely related (except for their degree of invasiveness) was important to minimize variables that would make the sample less homologous. We therefore sought paired osteosarcoma lines, where a more aggressive cell line was developed from a less aggressive ancestor without the use of exogenous transforming agents, as these agents may alter naturally occurring genetic changes leading to metastatic properties of osteosarcoma (Su et al., 2009). Without exogenous agents, we can attribute changes in cell morphology more directly to the difference in metastatic potential, as the *in vivo* development of the highly metastatic line more accurately represents the natural process of formation of metastases.

The morphology-related genetic changes that accompany transformation include both changes in cytoskeletal properties as well as changes in adhesive properties (Cavallaro and Christofori, 2001). We decided to use surfaces of different hydrophobicity in our experiments to explore this possibility as more hydrophobic surfaces are less amenable to protein deposition (Grinnell and Feld, 1982) and thereby are less favorable to cell adhesion than hydrophilic surfaces. We prepared three different glass surfaces of varying hydrophobicity (Fig. S1). These are glass detergent washed and air dried (GDA, contact angle 27.6°), glass acid etched and air dried (GAA, contact angle too small to measure), and siliconized ethanol treated (SET, contact angle 99°).

**Fig. 1.**
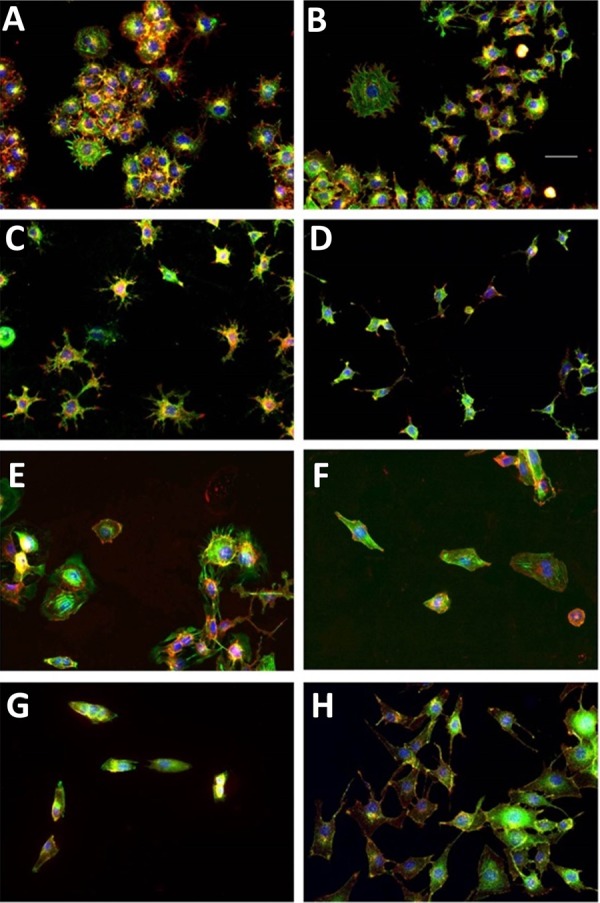
**Representative images of the four cell lines using fluorescence microscopy.** Each set of two panels represent the low metastatic (left) and the high metastatic (right) partner of a paired cell line. The cells nuclei (blue), the actin cytoskeleton (green) and the lipid membrane (red) of fixed cells are stained and are pseudo-colored as indicated for contrast. Note that the yellow color indicates the overlap of the red (membrane) and green (actin) channels. The cell lines are: (A) Dunn, (B) DLM8, (C) K12, (D) K7M2, (E) Saos2, (F) SAOS-LM7, (G) MG63 and (H) MG63.2. In all panels, scale bar is 50 μm.

We cultured four paired osteosarcoma cell lines with low and high metastatic potential: DUNN and DLM8; K12 and K7M2; MG63 and MG63.2; and Saos2 and SAOS-LM7 on these three surfaces for 48 h, and then fixed, stained and imaged the cells. For simplicity we refer to each pair by the first letter of the parental line, i.e. we refer to the pairs as the D, K, M and S pairs of lines. We stained the cells for actin, the plasma membrane and nucleus. We developed a high-throughput, quantitative image analysis algorithm that chose individual cells not in contact with others, segmented, optimized and thresholded the images to obtain accurate representations of two-dimensional shape and then processed the images to extract 29 morphometric measurements: 21 cellular and 8 nuclear (Table S1). Representative images of the eight different cell lines are shown in Fig. 1. Since here we are specifically looking for interpretable geometric differences, we did not consider other morphological representations such as shape representations in basis function expansions (Pincus and Theriot, 2007). We then subjected the data to statistical analysis to understand the differences between the high metastatic and low metastatic cell lines, using pairwise comparisons as well as by the multivariate principal component analysis (PCA) and nonmetric multidimensional scaling (NMDS). We developed a neural network machine-learning algorithm to try to distinguish between cells from the high metastatic and low metastatic cell lines.

## RESULTS

### Pairwise comparisons: the four paired cell lines demonstrated two distinct trends of cell shape changes

**Fig. 2.**
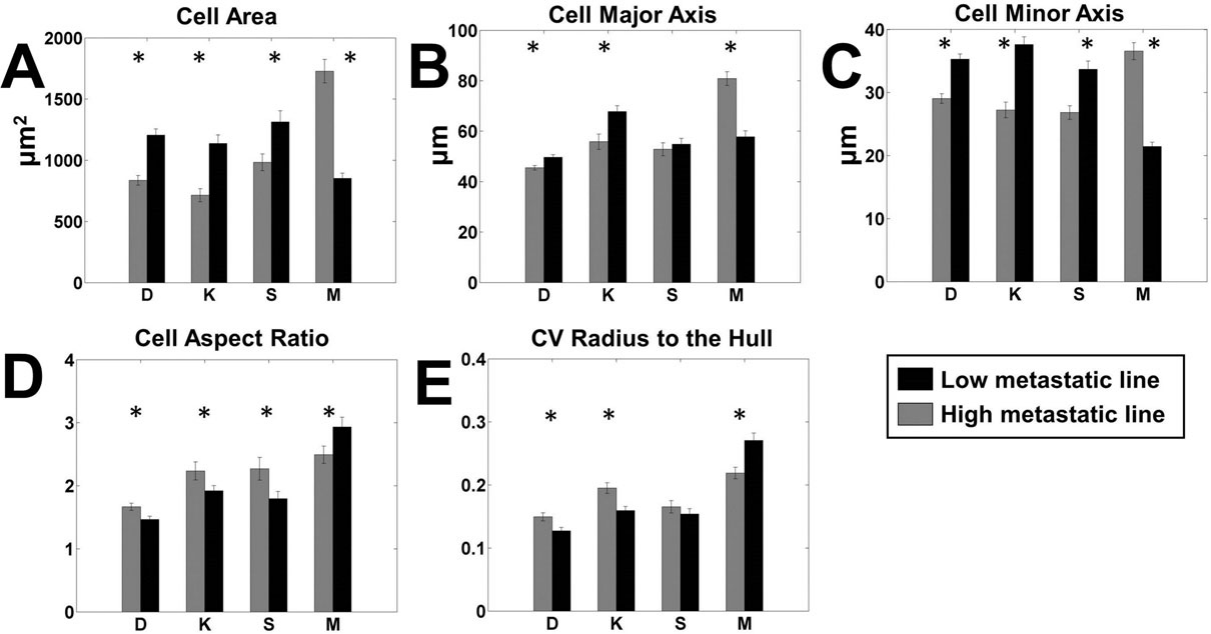
**Pairwise comparison of most significant cell shape parameters.** Each panel shows the comparison between high metastatic (grey) and low metastatic (black) cell lines for a single significant parameter on all surfaces. The paired lines are indicated by letters as follows. D: DUNN and DLM8; K: K12 and K7M2; S: Saos2 and SAOS-LM7; M: MG63 and MG63.2. (A) Cell area, (B) cell major axis, (C) cell minor axis, (D) cell aspect ratio and (E) coefficient of variation (CV) of the radius from the center of mass to the hull. *n*=100 for each cell line on each surface. *P<0.05 by two-tailed *t*-test satisfying the Holm–Bonferroni criteria for all variables (Table S3).

The 29 morphometric parameters were classified into five categories of cell shape: (i) projected cell size, (ii) cell roundness versus elongation, (iii) shape variability, (iv) nuclear size, and (v) nuclear shape. We identified a subset of the 29 parameters that were most often statistically significant across the various cell lines by performing pairwise *t*-tests between different morphometric measurements of the low metastatic line (low-met) and high metastatic line (high-met) within a paired cell line. In order to adjust for multiple testing, we performed *t*-tests on all 29 parameters using the Holm–Bonferroni correction (Benjamini and Hochberg, 1995), and identified the parameters that remain significantly different (Tables S2, S3). The data showed that metastatic cell lines show distinct differences in shape compared to their non-metastatic counterparts. Significantly, we discovered that three of the four paired cell lines, i.e. the D, K, and S lines displayed a similar pattern of shape changes, while the fourth line, i.e. the M line, displayed a different pattern (Fig. 2; Table S2). This suggests that morphological changes due to acquisition of metastatic potential fall into two distinct classes. For simplicity we denote these two patterns as type-1 and type-2. When pooled into two classes, the type-1 cells showed significant differences in 28 out of the 29 parameters we tested, while the type-2 cells showed significant differences in 26 parameters (Table S3).

### Highly metastatic cancer cells differ in cell volume and projected cell area

A striking gross morphological difference between the high-met and low-met line of each pair is a systematic difference in two-dimensional projected area. Less metastatic type-1 cell lines have a significantly larger projected cell area (Fig. 2A), and on average, the type-1 high metastatic lines are 30.7% smaller in area. The type-2 M lines showed the opposite trend, with highly metastatic cells being significantly more spread out, more than double the size of the parental line on some surfaces (Table S2). To determine whether cell volume corresponded with cell area, we measured cell volume using a handheld Scepter^®^ counter (Materials and Methods). We found that for most pairs the cell volume and the cell area followed the same trend, i.e. the high-met line was smaller in both area and volume for two lines of type I (K and S), while the high-met M line was larger in volume and area than its less metastatic pair. The D line showed an opposite trend with a large volume but smaller area for the high-met line. However the percentage difference in mean volume is much smaller than the percentage change in mean area, suggesting a difference in the spreading behavior of the cells for both type-1 and type-2 cells. This trend of a smaller projected high-met line for type-1 cells and a larger projected high-met line for type-2 cells is consistent across all 12 measures of two-dimensional cell size (Table S2). Within the type 1 cells, the largest difference was shown by the metastatic K12 cells that were over 50% smaller on GDA while the smallest difference was shown by the LM7 cells which were about 23% smaller (Table S2).

The change in size is also anisotropic, as the type-1 high-met lines have a minor axis that is 22% smaller, while the major axis is only 10.5% smaller, thus the minor axis percent difference is about twice that of the major axis (Fig. 2B,C), indicating elongation of the high-met type-1 cells relative to the low-met cells. The smaller size and elongated shape of high-met lines in type-1 osteosarcoma pairs are consistent across all three type-1 lines. The type-2 M cell lines showed the opposite trend for the major and minor axes with the high-met line having a larger minor axis by 67% and larger major axis by 48%, respectively.

### Highly metastatic cells differ in roundness, elongation and variability of perimeter

The second category of cell shape assesses how round versus elongated the cell is, and is best represented by the aspect ratio. As suggested by the major and minor axis differences discussed above, the type-1 highly metastatic cell lines had a significantly larger aspect ratio than the low-met lines, indicative of cell elongation (Fig. 2D). On average, the type-I highly metastatic cells were about 19% more elongated than the low metastatic cells. The maximum change here were the LM7 cells on SET with a 60% increase in the aspect ratio, while the smallest were the DLM8 cells with just about 13% increase on SET surfaces.

Variability of cell shape was characterized by the cell shape parameters of solidity and the coefficient of variation of radii drawn either to the cell perimeter from the center of mass of the convex hull (CV Rad Hull) or from the center of mass of the bounding circle to points on the convex hull (CV Rad Circle). The highly metastatic type-1 cell lines had more variability in radii drawn to both the perimeter (Table S2; Fig. 2E) and the convex hull. Another interesting measure is the circularity of the perimeter, which measures the deviation of the average shape from that of a circle. Circularity of the cell perimeter is significantly different between the high-met and low-met type-1 lines, by a little over 37% on average (Table S2).

The type-2 M lines showed the opposite trend to the ones listed above. The low metastatic cells had an aspect ratio which was about 22% larger on the GAA surface, and about 15% larger overall. The type-2 low-met line also displayed greater variability in shape than the high-met line with the CV of the perimeter radius larger by about 19% on average and by about 25% on the GAA surface. Similarly the CV of the Hull radius was larger by almost 28% on average for the low-met line. The circularity of the low-met type-2 line was also larger than its high-met partner, in contrast to the behavior shown by the type-1 lines.

### Highly metastatic cell lines show shape differences in the nucleus

**Fig. 3.**
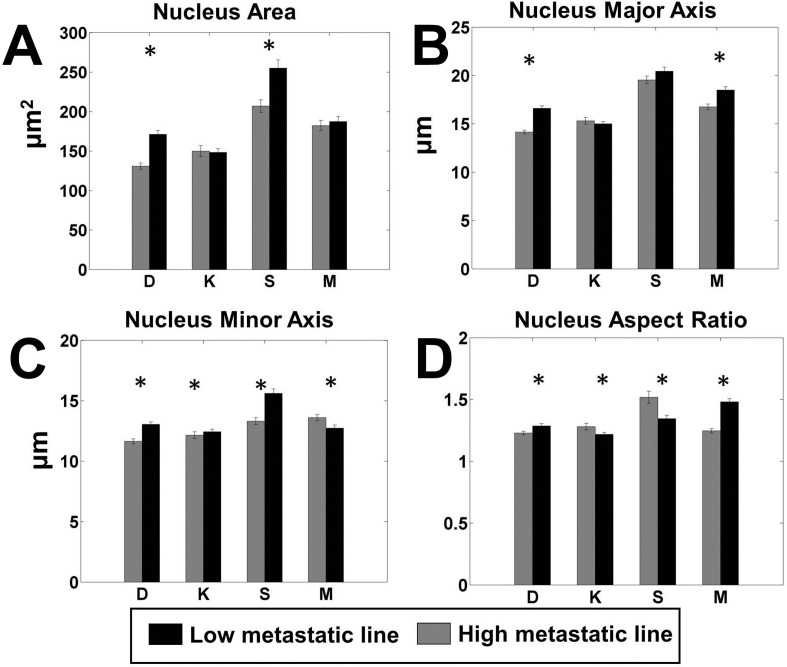
**Pairwise comparison of the most significant parameters of nucleus shape.** Each panel shows the comparison between high metastatic (grey) and low metastatic (black) cell lines for a single significant parameter. The paired lines are indicated by letters as follows. D: DUNN and DLM8; K: K12 and K7M2; S: Saos2 and SAOS-LM7; M: MG63 and MG63.2. (A) Nuclear area, (B) major axis of the nucleus, (C) minor axis of the nucleus and (D) aspect ratio of the nucleus. *n*=100 for each cell line on each surface. *P<0.05 by two-tailed *t*-test satisfying the Holm–Bonferroni criteria for all variables (Table S3).

Interestingly, the shape parameters of the nucleus also showed statistically significant differences between the high and low metastatic lines (Fig. 3). Nuclear size was larger for the low metastatic cells for all cell lines, including both type 1 and type 2. However while the larger nuclear size for low-met cells was statistically significant for the type-1 cells on GAA and SET surfaces, as well as for all surfaces, it was significant for the type-2 line only on the GAA surface. In line with the difference in nuclear area, the major and minor axes were larger for the low metastatic cells for all four pairs of cell lines (Fig. 3B,C). However, the nuclei aspect ratio showed mixed results, with the high metastatic lines demonstrating a larger aspect ratio for the D and M lines, while the low metastatic lines demonstrated a larger aspect ratio for the K and S lines (Fig. 3D).

This analysis also underscored the fact that every cell line contained a heterogeneous collection of cell shapes. The distributions of each parameter overlapped, which was not surprising given the fact that we chose the paired lines on the grounds that they were close to each other in genetic space. In the light of these results, we asked whether we could still see these differences using a multivariate measure by utilizing all the descriptors together.

### Multivariate Techniques show overlapping but distinct cell populations

**Fig. 4.**
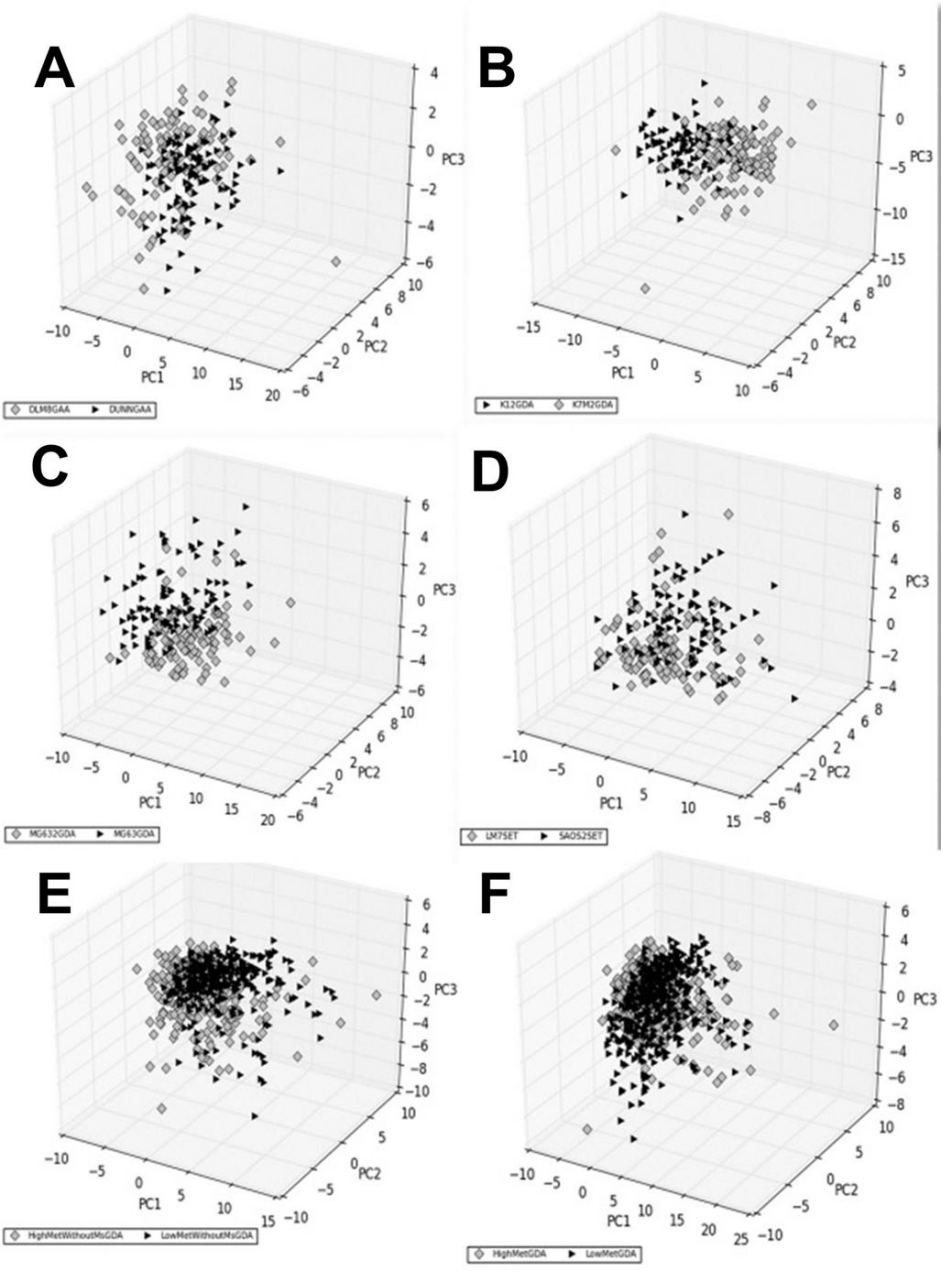
**Principal components of shape characteristics.** The shape characteristics data for each cell is projected onto the first three principal components of the combined data of each comparison. In this figure, comparisons for each paired cell lines that performed best are shown, as determined by visual inspection and global comparisons. The grey diamonds represent the high metastatic cell line(s) while the black triangles represent the low metastatic line(s). Each panel represents one comparison as follows: (A) DUNN vs DLM8 on GAA, (B) K12 vs K7M2 on GDA; (C) MG63 vs MG63.2 on GDA; (D) Saos2 vs SAOS-LM7 on SET; (E) all type-1 low metastatic lines versus high metastatic lines on GDA and (F) all low metastatic versus high metastatic (i.e. both types combined) on GDA. *n*=100 for each cell line on each surface.

We performed a principal component analysis (PCA) of the multivariate data, comparing each paired line separately (Fig. 4; Fig. S3). The PCA showed that the geometric characteristics of each cell type were overlapping but clustered distinctly within the space formed by the first three principal components. The overlap between the characteristics of the paired cell lines indicates that the high-met line is still not too dissimilar from the low-met line. However the genetic changes that accompany the acquisition of invasive characteristics have also resulted in the cell shape parameters drifting away from that of the original cell. The maximum overlap of the first three principal components can be seen in the SAOS-LM7 and Saos2 pair (Fig. 4D). The type-1 cells collectively show distinct clustering of the low-met and high-met populations (Fig. 4E), which is lost when we club the type-1 and type-2 cells together (Fig. 4F).

**Fig. 5.**
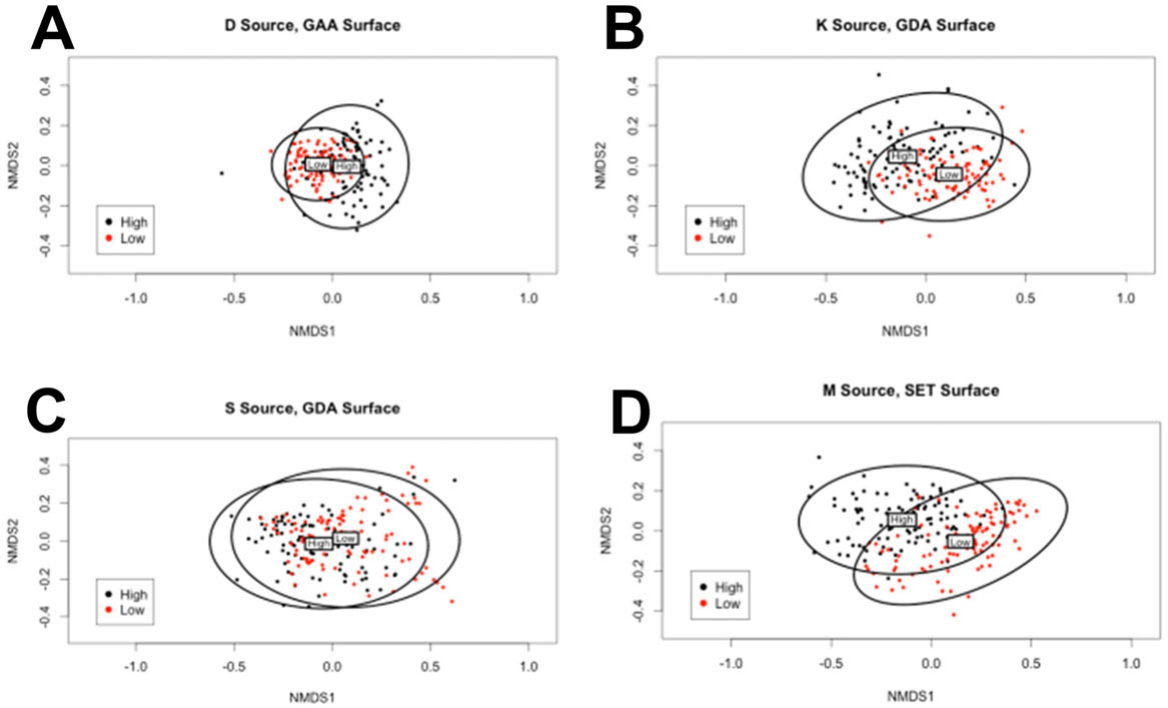
**Nonmetric multidimensional scaling.** Each panel represents an ordination pattern formed by comparison of geometric characteristics of a low metastatic and a high metastatic cell line on the surface that showed the highest R^2^ value for the pair. Each point represents the shape parameters of a single cell, plotted in black if high metastatic and red if low metastatic. The ellipses represent 95% confidence intervals with the labels ‘High’ and ‘Low’ marking the centroid positions of the corresponding cell line. The comparisons are as follows: (A) DUNN (low) and DLM8 (high) on GAA; (B) K12 (low) and K7M2 (high) on GDA; (C) Saos2 (low) and SAOS-LM7 (high) on GDA and (D) MG63 (low) and MG63.2 (high) on SET. *n*=100 for each cell line on each surface.

To test whether we could obtain a better separation using a nonlinear technique, we supplemented PCA by nonmetric multidimensional scaling (NMDS) (Anderson, 2001). NMDS is an ordination technique that seeks to find the ‘best’ coordinates for representing multivariate data in a lower k-dimensional ordination space. It does so by assessing and optimizing the agreement between ranked distance between data vectors in the original higher-dimensional space and the corresponding distance between them in k-space. Departure from this agreement is formally measured as ‘stress’. Other groups have used multidimensional scaling (MDS) to visually separate subpopulations of mesenchymal cells, further using this analysis to predict the fate of differentiating stem cells (Treiser et al., 2010). We used permutational multivariate analysis of variance to obtain the R^2^ values, where in the NMDS context R^2^ is a measure of the proportion of the distance variation of the data that is explained by cell line, i.e. from the high-met or low-met comparison within each paired line. Fig. 5 shows the NMDS results for the best-performing surfaces, and shows that the geometric characteristics overlap between paired lines but nevertheless cluster distinctly. The R^2^ values are tabulated in Table 1 along with their *P*-values. The maximum proportion of the distance variation that can be attributed to cell line is 0.16 for the D lines on the GAA surface, 0.2 for the K lines and 0.06 for the S lines (both on the GDA surface), and 0.24 for the M lines on the SET surface (0.22 on GDA). All the R^2^ values are statistically significant and indicate that cell shape parameters of the high-met line, despite significant overlap, have diverged from those of the low-met line. Other surfaces show varying levels of overlap but in general support this conclusion (Fig. S4). Interestingly data points corresponding to the high-met line for both type-1 and type-2 cells occupy a greater area in 2-space, suggesting that the high-met lines are characterized by greater heterogeneity of the shape parameters.

**Table 1.**
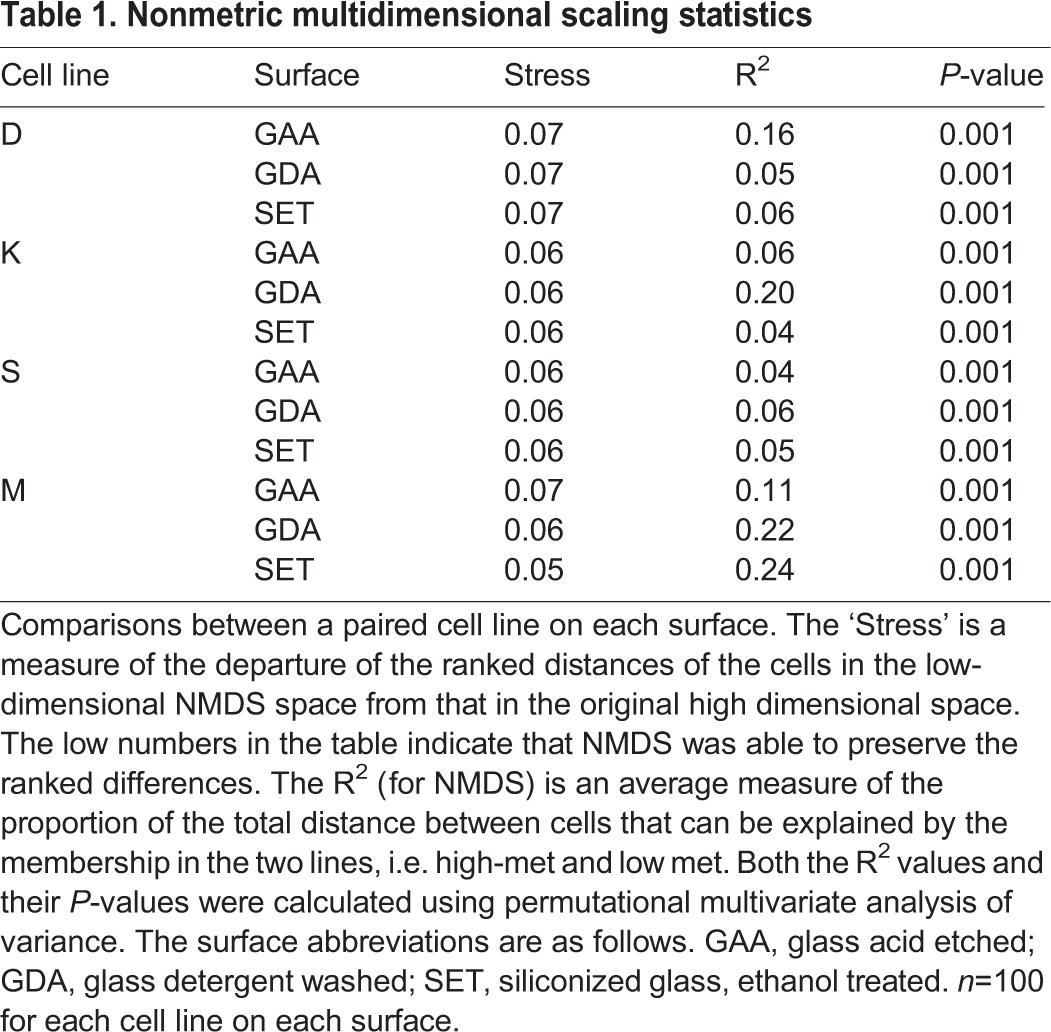
**Nonmetric multidimensional scaling statistics**

### Identification of cells by machine learning

We asked whether these subtle but significant differences in cell shape are sufficient to construct a classification algorithm that could correctly classify the low-met and high-met cells. We wrote a neural network machine-learning algorithm to classify a cell into either the low-met or the high-met class, based on its geometric parameters alone, as described in the Materials and Methods section. Following standard practice we divided our data into three mutually exclusive subsets for training, optimizing and validating the neural network respectively. The trained algorithm was then tested blind on the third subset, the validation set, which was not used for any parameter adjustment.

The accuracy of classification of the algorithm was found to lie between 60% and 92%, (Table 2) suggesting as high as about 40% and as low as 8% overlap of parameters. The latter figure is much lower than expected from the preceding analysis, probably due to the efficiency of the neural network in picking up subtle differences. Single cells from all the four lines can be classified with at least 80% accuracy on at least one surface.

**Table 2.**
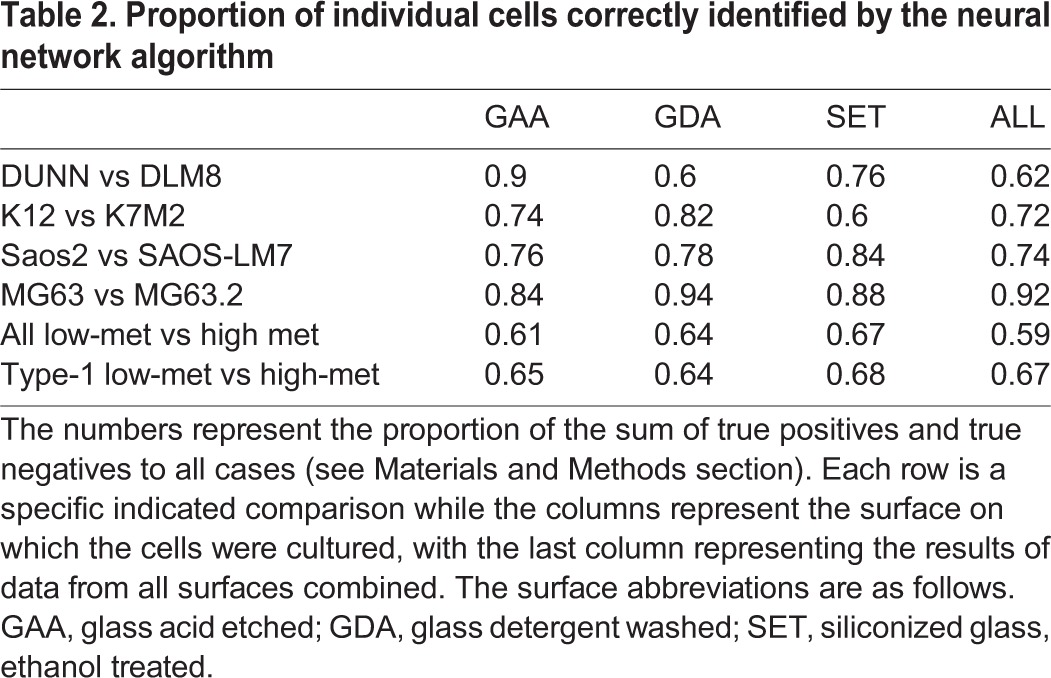
**Proportion of individual cells correctly identified by the neural network algorithm**

Next we asked whether the classification algorithm is capable of accurately classifying random samples of cells from the high-met and the low-met lines. This process can be construed as a simulation of what would happen in a clinical setting: the heterogeneous cancer cell population taken from a tumor biopsy or aspirate would be assayed using morphometric characteristics. The decision algorithm used was that if the majority of cells in the sample are of type A, the sample is of type A, and with this simple rule the algorithm achieves near perfect classification of samples into the correct cell type (Table 3). For every line there is at least one surface where samples can be classified with greater than 95% accuracy. Even for the S-line, where NMDS revealed only a 6% maximum difference between the cell line parameters, the neural network achieves a maximum classification accuracy of 99%.

**Table 3.**
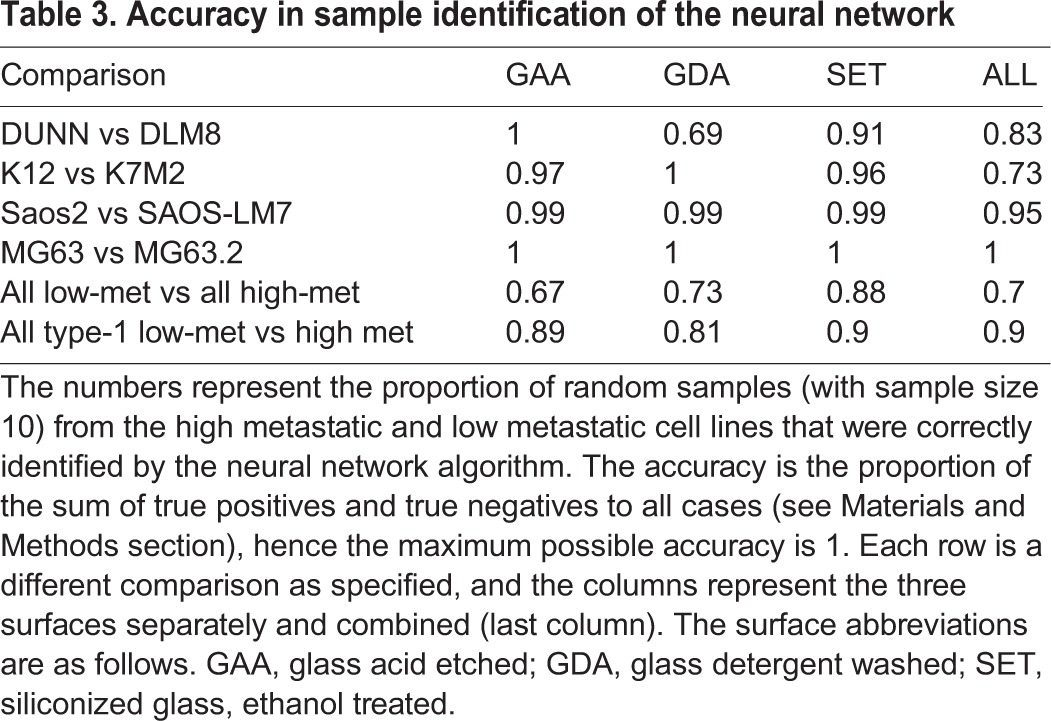
**Accuracy in sample identification of the neural network**

Note that the algorithm performs relatively poorly when used to classify samples from all low-met lines against all high-met lines as compared with when it is used on type-1 cells and type-2 cells separately. Thus, shape changes in the three paired lines in type-1 appear similar enough that despite originating from different species and different cell lines, they can be accurately classified into high-met and low-met cells with reasonable accuracy.

## DISCUSSION

We have shown that highly metastatic osteosarcoma cell lines derived from less metastatic parental cells show differences in shape that can be broadly classified into two types. In type-1, displayed by 3 out of 4 paired cell lines, the highly metastatic cells are smaller in two-dimensional area, more elongated, and the radius of the cell perimeter from the center of mass is more variable. In type-2 cells, displayed by one cell line pair, the cells become larger, more rounded, with a less variable perimeter. In both cases, the distribution of the geometric characteristics that we measured was more diverse for the high-met cell line. There was a significant overlap between the parameters of the low-met and the high-met line. However use of multivariate data analysis techniques such as PCA and NMDS indicated that despite the overlap, the data points of the two cell lines clustered slightly differently from each other. The differences were sufficient to enable a trained neural network to correctly classify an individual cell as belonging to the high-met or low-met line with over 80% accuracy on at least one surface, and to almost perfectly classify samples of cells from either line.

Our data suggest that genomic changes leading to acquisition of invasive properties also give rise to detectable shape changes, and hence shape changes carry information about the state of the cell. While this study was restricted to these four pairs of osteosarcoma cell lines, we suspect that the broad conclusions are more general. Genetic changes that drive the acquisition of invasive properties may affect cell shape in various ways. For example, there could be down-regulation of adhesive proteins, a softening of the cell due to down-regulation of keratin and up-regulation of vimentin and changes in cellular contractility due to Rho-ROCK signaling. Each of these is likely to have a different set of effects on cell shape, and requires further investigation. Identifying and understanding the full typology of shape changes could have a major impact on our knowledge of metastasis and its relation with the cellular cytoskeleton. It may be eventually possible to read out genetic changes corresponding to specific changes in cell shape (Bakal et al., 2007). Determining the causal links between genetic changes and the shape of the cell are outside the scope of the present paper (and are future goals), but we provide evidence that these links exist since functional changes in invasive properties correlates with changes in cell shape.

Our discovery that shape changes fall into two types or classes is also potentially significant. Our hypothesis arising from this work is that these two classes correspond to the two modes of cell migration, i.e. mesenchymal and amoeboid (Wolf et al., 2003). Mesenchymal motion consists of cell polarization, extension, substrate binding followed by actin-based contraction and release of focal adhesions at the trailing edge. This kind of migration is dependent upon adhesion receptors, as well as on the expression of enzymes that degrade the extracellular matrix such as MMPs (Wolf et al., 2003). However when enzyme activity of MMPs is blocked, cells are found to move in an amoeboid manner, wherein the cell squeezes itself into the empty spaces in the extracellular matrix. The two modes of motion are associated with different morphologies, with the mesenchymal mode corresponding to an elongated morphology and the amoeboid mode corresponding to a rounded morphology (Sanz-Moreno and Marshall, 2010). Recent studies have shown that the amoeboid mode of migration is associated with Rho signaling through ROCK and requires the protein ezrin, which links the cell membrane and the cellular cytoskeleton (Sahai and Marshall, 2003). Thus downregulation of MMPs and upregulation of ezrin appears associated with amoeboid motility. The highly metastatic MG63.2 line was found to be characterized by downregulation of MMPs and upregulation of ezrin (Su et al., 2009), suggesting that its preferred mode of motility could be amoeboid and providing an explanation for the rounded morphology it possesses as compared with the parent MG63 line. This suggests that cancer cells may acquire intrinsic preference for one mode of motility over the other as they acquire invasive characteristics, even if they are capable of switching modes of motility (Liu et al., 2015; Wolf et al., 2003). Thus type-1 cells could have an intrinsic preference for mesenchymal motility while type-2 cells could have an intrinsic preference for amoeboid motility.

Our work suggests that it is possible to develop a consistent reproducible framework for computational morphometrics of cell shape. Further work is required to validate and refine the framework through use of other cell lines, including primary tumor lines, other cancer types and species. A reproducible quantitative framework is important for improving the subjectivity of traditional morphological analysis performed by trained histopathologists. While there is a strong correlation between tumor grade and metastatic outcome, there is not yet an ability to predict metastatic potential, based on tumor grade, in individual cases (Loukopoulos and Robinson, 2007). One study found low reliability of the grading of chondrosarcomas, despite the fact that grading scores guide therapeutic decision-making (Eefting et al., 2009). A summary of numerous studies on the reliability and reproducibility of urologic, prostrate or renal cell cancer grading found low agreement and reproducibility (Engers, 2007). Our work provides some evidence that computational image processing based morphometry to assess tumor grade may help overcome some of these challenges.

A small number of recent publications have highlighted the functional importance of cell shape by using high throughput image analysis to characterize the relation between cellular morphology and cellular properties. Treiser et al. (2010) used quantitative morphometric descriptors along with MDS to predict differentiation of mesenchymal stem cells along bone or fat lineages at an early time point. They showed that subtle genetic differences between cells proceeding down the two lineages could be inferred from looking at small changes in cellular morphometrics. Yin et al. (2013) utilized high throughput imaging and computational methods to classify *Drosophila* haemocyte cells into five discrete shapes based upon quantitative shape and morphology metrics, and argued that transitions between these shapes are switch-like. They utilized RNAi to identify genes which play a large role in regulating cell shape, including demonstrating that the loss of PTEN induces elongation of cells. They did not however look for systematic differences between closely related cancer cell lines. While we have not tried to ascertain whether specific types of shapes are present in our data, the message of this paper is that differences in quantitative shape parameters, even within the same type, should carry useful information about the internal state of the cell. The overlap between the multidimensional shape parameters in principal component space or in NMDS space indicates that each pair of the cell lines we study has not diverged significantly in shape characteristics. However both these studies support our contention in this paper that the understanding of cell shape can give significant insight into cell properties and function.

Studies of metastasis in cancer cells have focused mainly on changes at the level of gene, protein and microRNA expression, and to a smaller extent, at the level of cellular mechanics. In contrast our work demonstrates that these changes do lead to reproducible changes in shape. More work needs to be done to construct a more comprehensive typology of shape changes in cancer, especially in other cancer types, and to achieve a mechanistic understanding of how changes in gene and protein expression result in changes in cell shape.

## MATERIALS AND METHODS

### Cell lines and cell culture

We utilized four paired cell lines; two of murine origin: DUNN and DLM8, K12 and K7M2, and two of human origin: MG63 and MG63.2, Saos2 and SAOS-LM7. All metastatic lines (DLM8, K7M2, SAOS-LM7, and MG63.2) have significantly higher rates of pulmonary metastasis reported in the literature with a 200-fold increase in MG63.2, 100% efficacy of DLM8 relative to no pulmonary metastases in DUNN, 100% efficacy of SAOS-LM7, and a 90% efficacy of K7M2 relative to 33% of K12. Additionally, MG63.2, DLM8, SAOS-LM7 and K7M32 cells were reported to show greater migration and invasion than their low-metastatic counterparts: MG63, DUNN, Saos2 and K12 (Asai et al., 1998; Jia et al., 1999; Khanna et al., 2000; Su et al., 2009). MG63.2 is reported to have weaker heterotypic adhesion than MG63, while K7M2 have higher initial rates of adhesion but no difference in ultimate adhesion (Khanna et al., 2000; Su et al., 2009).

DUNN, DLM8, K12, and K7M2 cell lines were a gift from Dr D. Thamm (Colorado State University, CO, USA), MG63 and MG63.2 cell lines were a gift from Dr D. Duval (Colorado State University, CO, USA), and Saos2 and SAOS-LM7 a gift from Dr E. S. Kleinerman (MD Anderson Cancer Center, TX, USA). All cell lines were maintained under typical culture conditions at 37°C and 5% carbon dioxide concentration in Dulbecco's Modified Eagle Medium (DMEM) (Sigma). DMEM was supplemented with 10% fetal bovine serum (Atlas Biologicals), 20 mM Hepes (Sigma), and 100 Units/ml penicillin with 100 µg/ml streptomycin (Fisher Scientific-Hyclone). Cell lines were not independently authenticated or tested for contamination by us.

### Immunofluorescence microscopy

Cells were cultured on indicated substrate for 48 h. Cells were stained with Wheat Germ Agglutinin, Alexa Fluor 594 Conjugate (Molecular Probes). Cells were fixed in 4% paraformaldehyde then stained with Alexa Fluor 488 Phalloidin and DAPI (Molecular Probes). Cells were imaged under a 20× objective on a Zeiss Axioplan 2 fluorescence microscope (Zeiss, Thornwood, NY, USA) using filter sets: DAPI BP 445/50 blue filter, HQ Texas Red BP 560/40, and Green BP 474/28.

### Preparation of surfaces

Three different surfaces were prepared for this work from either a #1.5 22 mm×22 mm glass coverslip (Richard Allen Scientific) or a #2 22 mm×22 mm siliconized glass coverslip (Hampton Research). The formulated surfaces follow: glass detergent washed and air dried (GDA), glass acid etched and air dried (GAA), and siliconized ethanol treated (SET). GAA and GDA surfaces were initially prepared by sonication for 30 min in a mild detergent solution. Following sonication, the coverslips were sequentially rinsed with Milli-Q (MQ) water, isopropyl alcohol (IPA), and a second rinse with MQ water prior to any further downstream processing. In the case of the GDA surface, no further downstream processing was required and the surfaces were blown dry with sterile 0.2 µm filtered nitrogen with an air gun from an in house boil off nitrogen source. GAA surface was subjected to a downstream 1 M hydrochloric acid etching at 60°C for 12-16 h. After the etching period, the coverslips went through the same rinse process described above (MQ to IPA back to MQ) before being blown dry in the same manner as the GDA surface. The SET surface was subjected to a rinse in 100% ethyl alcohol and then they were blown dry with the nitrogen gun to ensure removal of any residual liquid and debris. Prior to use in cell culture, all surfaces were exposed to UV sterilization to minimize potential contamination risks.

### Contact angle measurements

Contact angles for different substrates were measured using sessile drop method by Rame Hart Goniometer (Model # 100_25_M). 3 µl of Milli-Q water were placed on *XYZ* plane using needle. Images were captured and analyzed using Rame Hart DROP Image Advanced software. Contact angle were measured for three different spot on one slide and this was repeated three times on different slides to see variability of slide's contact angle. Representative images are shown in Fig. S1.

### Cell volume measurement using Scepter cell counter

Volume measurements were made by the Scepter™ Handheld Automated Cell Counter, Millipore, with a 60 µm sensor. First, cells were plated in a culture dish. Once they were ready to be split, they were trypsinized and re-suspended in 1× PBS. After checking that cell density was in the operating range (10,000-500,000 cells/ml) the Scepter sensor was submerged in the cell suspension. The upper and lower gates were adjusted to remove debris information, and cell volume information recorded.

The distributions of cell volumes were well approximated by a log-normal distribution. Thus we log-transformed and calculated the mean and the standard error of the mean of the resulting normal distribution. The mean cell volume for the cell lines were 1.01, 1.12, 1.60, 1.41, 1.49, 1.43, 1.66, 1.80 pl for DUNN, DLM8, K12, K7M2, Saos2, SAOS-LM7, MG63 and MG63.2, respectively. Thus the percentage differences between the volume of the high-met line from the low-met line are −10.9% (D lines), +11.9% (K lines), +4% (S lines) and −8.4% (M lines). We performed *t*-tests against the null hypothesis that the volume data for both pairs of each paired line came from a distribution with the same mean. The mean volumes for the two partners in a paired line were statistically different from each other, with *P*-values much smaller than 0.05 in each case.

### Image processing

Images of isolated cells that were not in contact with other cells were chosen. To ensure adequate statistical power we picked a sample of 100 such cell images (so that the power of the test for comparing means would be 80% at 1% significance level for a half-standard deviation effect size). Images were collected blind in the sense that the students doing the imaging were not previously aware of any differentiating characteristics discovered. The images were collected at one time for each cell line on each surface. The image processing involved three distinct steps; enhancement, conversion into binary format, and automated cropping of each cell for measurement of shape metrics. Three channels were captured as described above. Prior to processing the images were converted into 16 bit TIFF images. The exported TIFF images were loaded into MATLAB where the actin, membrane, and nuclei channels were enhanced separately by contrast stretching. The actin and membrane images were combined into a single TIFF images to get full characterization of the shape. Finally, erosion with a three pixel mask was applied to sharpen edge boundaries. The enhanced TIFF images of the combined membrane-actin image and separate nuclei image were exported into ImageJ analysis for manual conversion into binary formatted images by thresholding (a representative example is shown in Fig. S2). Once the images had been converted into binary format, they were again loaded into MATLAB for shape analysis. Segmentation was achieved through use of the built-in MATLAB function toolbox so each cell could be individually cropped and reconstructed in a new image in which shape measurements (listed in Table S1) could be made on both the cell and corresponding nuclei and stored for statistical analysis. Minimum Bounding Circle was found using MATLAB function minboundcircle, open source code developed by John D'Errico (http://www.mathworks.com/matlabcentral/fileexchange/34767-a-suite-of-minimal-bounding-objects/content/MinBoundSuite/minboundcircle.m). Scripts for image processing will be made available upon request.

### Data analysis

#### *t*-test

Individual cell metrics were compared as discussed in the results section utilizing the built in MATLAB ttest2 function which returns a test for the null hypothesis that the data come from independent random samples with normal distributions and equal means without assuming equal variance. This is a two-tailed test. The null hypothesis is initially rejected at a 5% significance level. All 29 parameters are then retested with the significance level determined by the Holm–Bonferroni correction for multiple tests.

#### Principal component analysis (PCA)

PCA is a method to project each sample in specific dimension to a space with equal or smaller dimension. This process is done in such a way that the first principal component has the maximum variance, second principal component has the next maximum variance, and this rule continues for subsequent components. The principal component vectors also form an orthogonal basis. We used singular value decomposition (SVD) to perform PCA on the data. First, data was standardized so that mean of new data is zero and standard deviation is 1. Then, SVD of the data was computed and the principal components extracted from the right singular vectors of the data. Each data point was then projected into the space formed by the first three principal components, and was plotted for visualization. The variance captured by the first three principal components lie in the range 44%-47% of the total variance for every comparison made.

#### Nonmetric multidimensional scaling (NMDS)

We performed separate analyses for each of the three surfaces (GAA, GDA, SET) and each paired cell line (D, K, S and M). First, each of the 29 cell morphology variables was relativized by dividing each value by the maximum value. Statistical software R (version 3.1.2) and package vegan were used to perform all statistical analyses. The Bray–Curtis dissimilarity index was used to perform NMDS. Based on observed stress, convergence behavior, Shepard plots, and parsimony, k was chosen to be 3.

The ordination pattern was scaled as follows before plotting. First, centering was done to move the origin to the average of the axes. Second, principal components were used to rotate the configuration so that the variance of points was maximized for the first dimension, with the second dimension explaining the maximum variance of points unexplained by the first. We then displayed the ordination pattern in 2-space. For the factor ‘Metastatic capacity’ (with levels Low and High) and the factor ‘cell line pair’ (with levels D, K, M, and S), we generated two separate color-coded plots with 95% confidence ellipses and labeled locations of the level centroids. For GAA, GDA, and SET, observed stresses were approximately 0.07, 0.07, and 0.08.

Permutational multivariate analysis of variance using Bray–Curtis distance between cells (PERMANOVA) was used to obtain R^2^ values for ‘metastatic capacity’. Specifically, R^2^ is a measure of the proportion of the data (distance) variation explained by ‘metastatic capacity’.

#### Machine learning

A multilayer perceptron (MLP) neural network with one hidden layer, adapted with permission from a version used by Dr Charles Anderson for teaching (http://www.cs.colostate.edu/∼anderson/cs545/index.html/doku.php), was used to classify data, and is available from the corresponding author upon request. A back-propagation learning algorithm, which uses a scaled conjugate gradient (SCG), was used to design the MLP and tanh(x) was used as the activation function. The SCG was adapted from Nabney's netlab library (Møller, 1993; Nabney, 2002). Each data set was partitioned into test, training and validation data at 50%, 25%, and 25% of data respectively. The test and training data sets were used to find the best attribute combinations, number of hidden units and weight parameter values in the non-linear logistic regression model. Initial parameters are chosen randomly. Training data was used to fit parameters by maximizing a likelihood function; testing data was then used to calculate the percentage of cells classified correctly (test percent). To optimize the model, training and test data were repartitioned and an average test percent was calculated for different attribute combinations and function structure; we selected the optimal attribute combination and function structure based on the maximum average test percent. After the function structure was chosen the test and training data sets were combined for one last round of optimization of the weights. The optimized model was then used to predict the class that each individual cell in the validation data belongs to with no further adjustment of parameters.

To test the accuracy on random samples of cells from each population, after identifying the function structure with the training and test data, we took 100 random paired samples of 10 cells each from the validation data set. The percentage of cells in each sample predicted to be class 1 are recorded (*P*). Thus the percentage of cells predicted to be class 2=1−*P*. A decision threshold was determined utilizing the false negative rate (FNR) and true positive rate (TPR). When *P* was bigger than the decision threshold, the sample was classified as class 1, and when it was smaller than the decision threshold as class 2. As detailed in Table S4, the threshold was optimal at 0.6. From the total 100 pairs, the true positive (TP), true negative (TN), false positive (FP) and false negative (FN) were calculated. Using this information, accuracy, false negative rate (FNR) and true negative rate (TPR) were calculated as defined below: